# The Evaluation of Telehealth's Impact on Medicare Annual Wellness Visits and Dementia Diagnosis

**DOI:** 10.1002/alz70863_110676

**Published:** 2025-12-23

**Authors:** Zhang Zhang

**Affiliations:** ^1^ Department of Health Policy and Management, Johns Hopkins Bloomberg School of Public Health, Baltimore, MD USA

## Abstract

**Background:**

Telehealth has emerged as a pivotal tool in modern healthcare and holds particular promise for early screening and diagnosis of cognitive impairment and dementia, which are critical for timely interventions. A key initiative in this area is the digitalized Montreal Cognitive Assessment, a sensitive screening tool for detecting executive dysfunction. MoCA can be integrated into annual wellness visits (AWVs), which are free benefits that screen for cognitive impairment and develop personalized prevention plans among Medicare beneficiaries in the U.S. Telehealth has the potential to improve access to AWVs, streamline cognitive assessments, and subsequently facilitate earlier dementia diagnosis. However, barriers to telehealth adoption remain unclear for vulnerable populations, such as older adults with risk factors for Alzheimer's Disease and Related Dementias. This study aims to evaluate the impact of telehealth on AWV uptake and the modality of AWV completion (in‐person vs. telehealth).

**Method:**

We created a longitudinal cohort using 2020–2022 Medicare Advantage (MA) and traditional fee‐for‐service (FFS) data. We applied probit regression models to examine the relationship between telehealth adoption and AWV uptake. An instrumental variable (IV) design was used to address potential endogeneity. Geographic clusters of internet connectivity (e.g., broadband) and county‐level telehealth adoption rates among providers served as IVs to predict telehealth use. We included beneficiaries aged 65 and older who were continuously enrolled in either MA or traditional FFS plans.

**Result:**

We found a significant increase in the likelihood of AWV uptake among telehealth adopters by 10.2 percentage points (*p* <0.001) than non‐adopters of telehealth in traditional FFS Medicare plans. We will examine the pattern among MA beneficiaries. We expect the effect will be more pronounced for MA beneficiaries than those in traditional FFS plans. This difference may reflect MA's greater ability to manage health expenditures and provide additional resources, such as internet access and incentives for preventative care, that improve AWVs via telehealth.

**Conclusion:**

Our study demonstrates the role of telehealth in increasing AWV uptake and facilitating cognitive assessments. Telehealth has the potential to bridge gaps in cognitive screening and enhance early detection of MCI and ADRD, supporting timely diagnosis and care planning.

